# Effects of the 2018 Japan Floods on long-term care insurance costs in Japan: retrospective cohort study

**DOI:** 10.1186/s12889-022-12492-7

**Published:** 2022-02-17

**Authors:** Shuhei Yoshida, Saori Kashima, Shinya Ishii, Soichi Koike, Masatoshi Matsumoto

**Affiliations:** 1 Department of Community-Based Medical System, Graduate School of Biomedical and Health Sciences, Hiroshima University, 1-2-3 Kasumi, Minami-ku, Hiroshima-ken, Hiroshima-shi, 734-8551 Japan; 2 Environmental Health Sciences Laboratory, Graduate School of Advanced Science and Engineering, Hiroshima University, 1-3-2 Kagamiyama, Hiroshima-ken, Higashi-Hiroshima-shi, Japan; 3 Department of Medicine for Integrated Approach to Social Inclusion, Graduate School of Biomedical and Health Sciences, Hiroshima University, Hiroshima, Japan; 4 Division of Health Policy and Management, Center for Community Medicine, Jichi Medical University, 3311-1 Yakushiji, Tochigi-ken, Shimotsuke-shi, 329-0498 Japan

**Keywords:** Climate change, Natural disaster, Disaster preparedness, Long-term care, Long-term care insurance service, Claim data, Rural health services

## Abstract

**Background:**

Climate change has increased the frequency and severity of torrential rains and floods around the world. Estimating the costs of these disasters is one of the five global research priorities identified by WHO. The 2018 Japan Floods hit western Japan causing extensive destruction and many deaths, especially among vulnerable elderly. Such affected elderly would need long-term care due to the various health problems caused by the disaster. A Long-Term Care Insurance (LTCI) system provides care services in Japan. The aim of this study was to evaluate the effect of the 2018 Japan Floods on LTCI costs and service utilization.

**Methods:**

The participants of this retrospective cohort study were all verified persons utilizing LTCI services in Hiroshima, Okayama and Ehime prefectures. The observation period was from 2 months before to 6 months after the disaster. We used Generalized Estimating Equations (GEEs) to examine the association between disaster status (victims or non-victims) and the monthly total costs of LTCI service (with gamma-distribution/log-link) by residential environment (home or facility). Among home residents, we also examined each service utilization (home-based service, short-stay service and facility service), using the GEEs. After the GEEs, we estimated Average Marginal Effects (AME) over all observation periods by months as the attributable disaster effect.

**Results:**

The total number of participants was 279,578. There were 3024 flood victims. The disaster was associated with significantly higher total costs. The AME for home residents at 2 months after was $214 (Standard Error (SE): 12, *p* < 0.001), which was the highest through the observation period. Among facility residents, the AME immediately after the disaster increased by up to $850 (SE: 29, *p* < 0.001). The service utilization among home residents showed a different trend for each service. The AME of home-based services decreased by up to − 15.2% (SE:1.3, *p* < 0.001). The AME for short-stay service increased by up to 8.2% (SE: 0.9, *p* < 0.001) and the AME for facility service increased by up to 7.4% (SE: 0.7, *p* < 0.001), respectively.

**Conclusions:**

The 2018 Japan Floods caused an increase in LTCI costs and the utilization of short-stay and facility services, and a decrease in utilization of home-based services.

**Supplementary Information:**

The online version contains supplementary material available at 10.1186/s12889-022-12492-7.

## Background

Climate change is an inseparable factor affecting human health and wellbeing [1]. The World Health Organization (WHO) reported that climate change is expected to cause approximately 250,000 additional deaths per year between 2030 and 2050 [2]. Due to the effects of climate change, the frequency and magnitude of disasters caused by floods or torrential rains have increased in recent years around the world [3]. Estimating the costs of these climate change-related disasters is one of the five global research priorities identified by WHO [4].

In Japan, torrential rains have occurred every year in recent times, with the 2018 torrential rains and floods in western Japan being the largest so far [5, 6]. Due to the severity of the storms, they became known as the 2018 Japan Floods (*2018-nen-sitigatu-gou*) by the Japan Meteorological Agency [7]. The enormous damage was reported as 237 dead, eight missing, 433 injured, and 6767 houses completely destroyed [8, 9]. The disruption of transportation networks and utilities made it difficult to transport necessary emergency relief supplies [10]. The amount of damage caused by the 2018 Japan Floods was approximately US$9.86 billion (\1158 billion), which was the largest amount of damage ever caused by a single incidence of torrential rains or floods in Japan [11].

Natural disasters often cause not only direct disaster-related deaths, but also post-disaster physical or mental problems [12–20]. Especially, disasters affect older people to a greater extent [21]. In the Great East Japan Earthquake (GEJE), most victims and deaths were among the elderly. Similarly, 90% of victims during the torrential rains were over 65 years of age [9]. Elderly victims often need long-term care as their health status becomes exacerbated [22]. Therefore, it is important to estimate the costs of long-term care caused by these disaster-related health problems.

To provide long-term care for the vulnerable elderly, the Japanese government established a public Long-Term Care Insurance (LTCI) system in 2000 [23–25]. The LTCI system provides necessary care services for the elderly. The main users of LTCI services are 65 years of age or older. The care services are broadly divided into three groups: 1) home-based services providing care while living at their private homes, 2) short-stay services that consist of respite care for a short period and 3) facility services that provide residence care to those who are unable to live at home [23, 26]. Local governments administer the LTCI system. Japan has the highest rate for an aging population among the world and currently more than 5 million people have received LTCI certification [27]. Therefore, the LTCI system has become indispensable for Japanese social security [23].

Although the increase of disaster-related LTCI costs is an urgent issue in an aging society, few studies have examined the effect of disasters on LTCI costs. Community-based ecological studies showed changes in LTCI service after the GEJE [28–30]. However, there are no studies that examine the ever-changing effect of a disaster at the individual level chronologically and precisely. In addition, since the impact of the disaster from torrential rains and floods is different from an earthquake, the impact on LTCI service could be different [31]. For these reasons, it is highly important to examine the impact of the 2018 Japan Floods on LTCI costs.

In this study, we investigate the effect of the 2018 Japan Floods on LTCI cost for disaster victims. Based on the results, we examined the impact of torrential rains or floods, which are increasing due to climate change, on the costs of elderly long-term care and provide insights for other countries considering the introduction of a public LTCI system.

## Methods

### Study design

This study was a retrospective cohort study.

### LTCI system

The utilization of LTCI services in Japan requires a certification of care-level. Elderly persons are certified after an application that they or their family submit. Subsequently, a care manager, a local official or other care-related professional that is independent from applicants are asked by the local government to visit the applicant to evaluate their care needs by using a structured questionnaire (*nintei-tyousa*). The certification also requires a physician’s written opinion (*shujii-ikensyo*), which is a care evaluation by a primary care physician. Next, a Care Need Certification Committee determines the care-level based on both evaluations. The care-levels are graded into 7 levels (support need levels 1–2 and care need levels 1–5). The higher the care-level, the higher the monthly limit for LTCI services. If the care needs change due to a disease or Activity of Daily Living (ADL), applicants can apply for a re-certification to change the care-level. The certification requiring support 1, which is the lowest care-level, allows for the use of services up to about US$455 (\50,000) per month. The person certified as requiring long term care 5, which is the highest care-level, can use LTCI services up to about US$3257 (\358,300) per month. Out-of-pocket expenditures for LTCI is from 10 to 30% according to income.

Various types of public or private residential facilities are involved in LTCI services. Each type of facility has a specific criterion for admission. Generally, a certain care-level is needed for admission to each facility. The daily basic cost for residential facility service is set per the facility type. Care for residents is provided within the basic cost.

### Data source

This study was conducted using a special sampling of certification data for long-term care and long-term care insurance claim data (Approval no. 0711–1). These data were stored in a long-term care insurance comprehensive database, which is managed by the Ministry of Health, Labour and Welfare (MHLW). This database collects digitized claims of LTCI that are summarized monthly with details for all services used by each user. This database also includes age classification, gender and other characteristics of users concerning long-term care insurance. The MHLW has provided datasets extracted from this database for research institutes since 2018, with the permission of an expert council. Researchers can apply for its use through the MHLW website and are allowed to use the data after meeting security systems such as limiting the member of data users [32].

If a LTCI user interrupts service use during a month due to any reason, such as hospitalization, death or change of needs, the cost for LTCI service does not accrue. The reasons are not included in this database.

### Setting

**Fig. 1:**
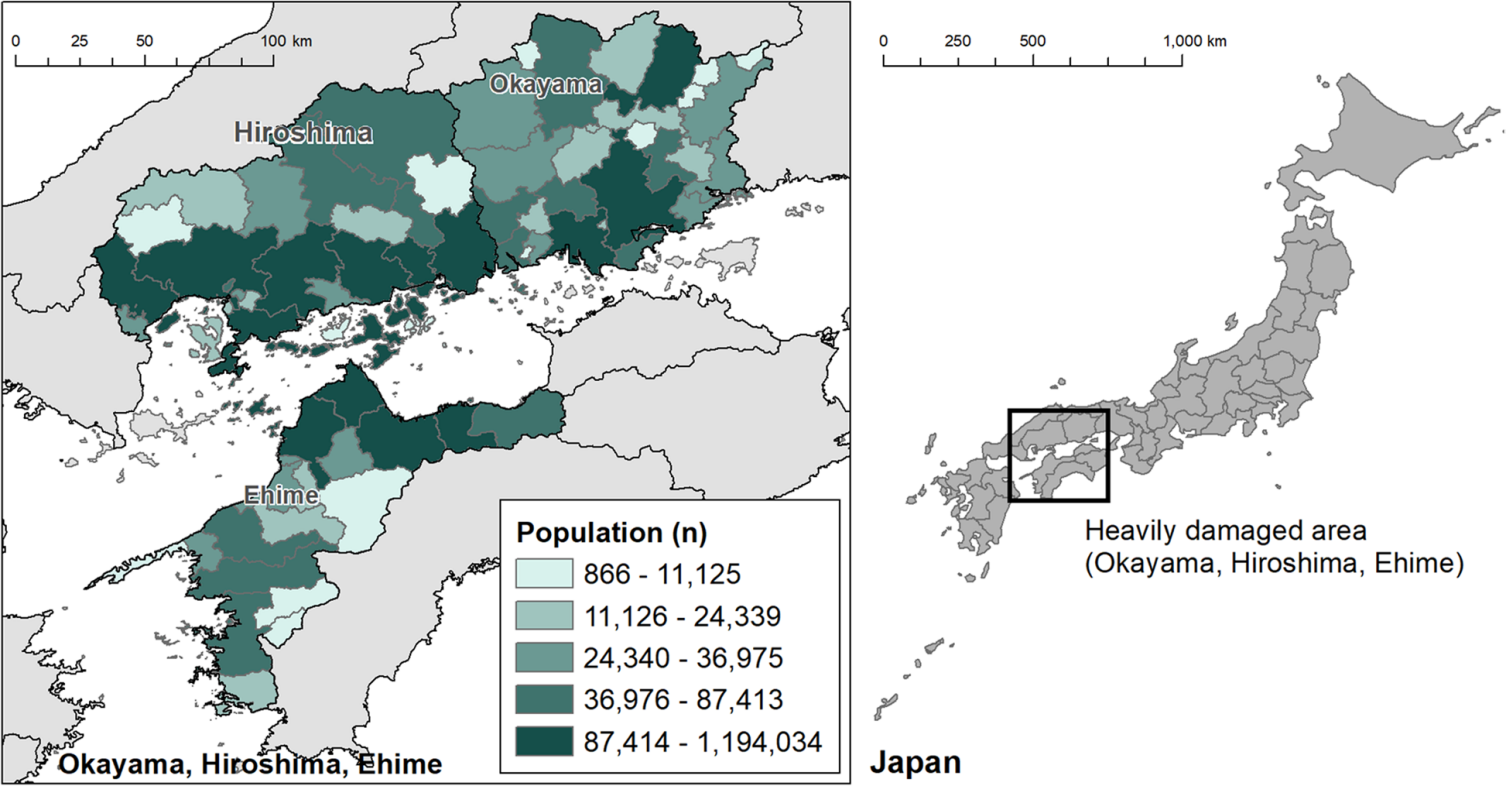
Population by municipality. Footnote: Population data was obtained from Statistical Observations of Municipalities 2020 published by the Ministry of Internal Affairs and Communications

The setting was Hiroshima, Okayama and Ehime prefectures (Fig. 1). These prefectures accounted for 212 out of 237 deaths, 8 out of 8 missing, 6603 out of 6767 houses completely destroyed and 10,012 out of 11,243 houses partially destroyed [9]. Because of the concentration of the damage, we choose these locales as the study setting.

### Participants

The participants were all LTCI service users in Hiroshima, Okayama and Ehime prefectures during May 2018 (2 months before 2018 Japan Floods). There were 368,778 certified people in these prefectures regardless of the service utilization of LTCI [33]. The observation period was from May 2018 to December 2018 (6 months after 2018 Japan Floods).

### Disaster status

We defined victims as participants whose monthly cost for LTCI services changed to exempt after the disaster. This conformed with the announcement of MHLW that all victims of this disaster were exempt from LTCI service costs, even if they used LTCI services in another region from their registered home region [34]. Local governments also authorized a designation as victim when a person’s house was completely or partially damaged, burned down, or flooding of a floor, or similar damage, when a main breadwinner died, the breadwinner was seriously injured or became ill, or the breadwinner went missing. Only 10 people were exempted for the LTCI service cost until the year before the disaster [35]. Because this exemption excluded those who paid no out-of-pocket expenditures for the LTCI service cost, such as welfare recipients and A-bomb survivors, we could not detect their impact by the disaster.

#### Covariates

We extracted the monthly total costs for LTCI services and the monthly utilization of each LTCI service type, disaster status (victims or non-victims), age classification, gender, care-level, residential environment (home residents or facility residents) and facility transfer. The monthly total costs for LTCI services were based on the amount of service which a participant used in a month. We converted Japanese yen to US dollars using the average rate for 2018 ($1 = \110) [36]. The LTCI service type was defined by Service Classification Code (*service-kubun-code*) and classified into three types: home-based service, short-stay service, and facility service (Supplementary Table 1 shows this in more detail [see Supplementary File 1]). We determined the utilization of each LTCI service type by the accrual of claim data. We defined home residents as users who did not use a facility service on the first month of the observation period (2 months before the disaster). For facility residents, a change in the facility code or the service classification code after the disaster was judged as a facility transfer.

#### Statistical analysis

We described the baseline characteristics of victims and non-victims. Chi-square test was used for the discrete variables. Wilcoxon’s rank sum test was performed for ordinal variables and for continuous variables without a normal distribution.

We used Generalized Estimating Equations (GEEs) to examine the association between disaster status and the costs of LTCI service. The dependent variables were the monthly total LTCI costs by home residents and facility residents. Since our primary interest was the monthly change due to the disaster, we added the interaction term between disaster status and month along with disaster status, month, age classification, gender and care-level as covariates. The GEE models were specified with a Gamma distribution and a log-linked function, because cost data usually skewed to the right. This model has been proven to be appropriate for analyzing cost data [37]. This approach was applied to the past studies when estimating costs [38–40]. However, a large proportion of zeros (20% or more) can cause inadequate estimation [18]. After we confirmed that the proportion of zeros by each month was less than 20%, we applied these GEE models.

The costs for facility residents are calculated comprehensively for all care provided in the facility. Meanwhile, the costs for home residents are calculated by adding various home-based services and short-stay services. Furthermore, home residents could move into facilities during the observation period. Therefore, we also examined the association service utilization among home residents and disaster status, using GEEs with the same covariates. When the incidence of an outcome is over 10%, the adjusted odds ratio derived from the logistic regression overestimates the risk ratio [41]. Because the incidence of service utilization is common (> 10%), the GEE models were specified with a Poisson distribution, a log-linked function and robust error variances [42, 43]. These modified Poisson models are robust when estimating relative risks or risk ratios for common binary outcomes [44]. Because the utilization of facility service did not accrue for the first month of the observation period (2 months before the disaster) as per our definition, we examined the utilization from 1 month before to 6 months after the disaster. Home-based service and short-stay service were examined through all observation periods. Because the incidence of outcomes is common (> 10%), the GEE models were specified with a Poisson distribution, a log-linked function and robust error variances [42, 43].

In the GEE models, we took into account correlations among individuals using exchangeable correlation structures. After conducting the GEEs, we used these models to estimate the disaster attributable impact by each month as Average Marginal Effects (AME). AME were estimated to assess the impacts on LTCI costs and the probability of LTCI service utilization associated with changes in disaster status, keeping all of the other covariates fixed.

We performed all statistical analyses using STATA/MP version 16 (StataCorp, 2019).

## Results

The total number of participants was 279,578. Victims, whose monthly cost for LTCI services changed to exempt after the disaster, accounted for 1.1% of the total (*n* = 3024).

Participant characteristics are shown in Table 1. The proportion of males was under 30% in both groups. The care-level of victims was lower than non-victims by Wilcoxon’s rank sum test (*p* < 0.001). Participants whose care-level changed during the observation period were 18.3% in victims (*n* = 550) and 15.1% in non-victims, respectively. This was statistically significant by chi-square test (*p* < 0.001). The proportion of facility residents at 2 months before the disaster was 22.2% (*n* = 672) in victims and 30.3% (*n* = 83,795) in non-victims. Participants who did not use LTCI services for an entire month during the observation period was 26.7% (*n* = 808) in victims and 16.0% (*n* = 44,181) in non-victims (*p* < 0.001). Among home residents, the total service cost of victims was lower than that of non-victims (*p* < 0.001). In contrast, among facility residents, that of victims was higher than that of non-victims (*p* = 0.003). Among facility residents, 36.7% of victims (*n* = 247) and 3.6% of non-victims (*n* = 3028) were transferred to different facilities after the disaster (*p* < 0.001).

**Table 1:** Demographic Characteristics

	Victims of the disaster		Non-victims of the disaster		*p* value
	*n* = 3024	1.1%	*n* = 276,554	98.9%	
Age, no. (%)					
under 65	59	2.0	5696	2.1	0.02*
65–74	314	10.4	26,908	9.7	
75–84	1012	33.5	86,869	31.4	
over 85	1639	54.2	157,081	56.8	
Gender, no. (%)					
Male	897	29.7	77,738	28.1	0.075*
Female	2073	68.6	192,987	69.8	
Missing^+^	54	1.8	5829	2.1	
Level of long-term care need, no. (%)					
Support need level 1‡	299	9.9	21,555	7.8	< 0.001†
Support need level 2	398	13.2	28,387	10.3	
Care need level 1	727	24.0	61,979	22.4	
Care need level 2	584	19.3	54,551	19.7	
Care need level 3	398	13.2	42,414	15.3	
Care need level 4	342	11.3	37,282	13.5	
Care need level 5	276	9.1	30,285	11.0	
Change in the level of long-term care need at December, no. (%)					
Increased	376	12.4	29,074	10.5	< 0.001*
Unchanged (without re-certification)	2047 (1881)	67.7 (62.2)	202,166 (155,845)	73.1 (56.4)	
Decreased	174	5.8	12,647	4.6	
Missing§	427	14.1	32,667	11.8	
Facility residents, no (%)	672	22.2	83,795	30.3	< 0.001*
Facility transfer, no (%)	247	36.7	3028	3.6	< 0.001*
Existence of a total disused month of LTCI service, no (%)					
All period	808	26.7	44,181	16.0	< 0.001*
Month					
1–3	502	16.6	24,940	9.0	< 0.001*
4–6	564	18.7	37,217	13.5	< 0.001*
Cost of per month, mean (SD)					
Home residents	1029	1029	1002	832	< 0.001†
Facility residents	2404	1219	2115	850	< 0.001†
Mean of LTCI service utilizations among home residents per month, no (%)					
Home-based service	1399	59.5	137,956	71.6	< 0.001*
Short-stay service	288	12.3	21,906	11.4	< 0.001*
Facility service	165	7.0	4513	2.3	< 0.001*

Footnote

*LTCI* long term care insurance, *SD* standard deviation

^*^chi-squared test

^†^Wilcoxon rank-sum test

^‡^Include project eligible persons, exclude them from the chi-squared test

^§^exclude them from the chi-squared test

**Fig. 2:**
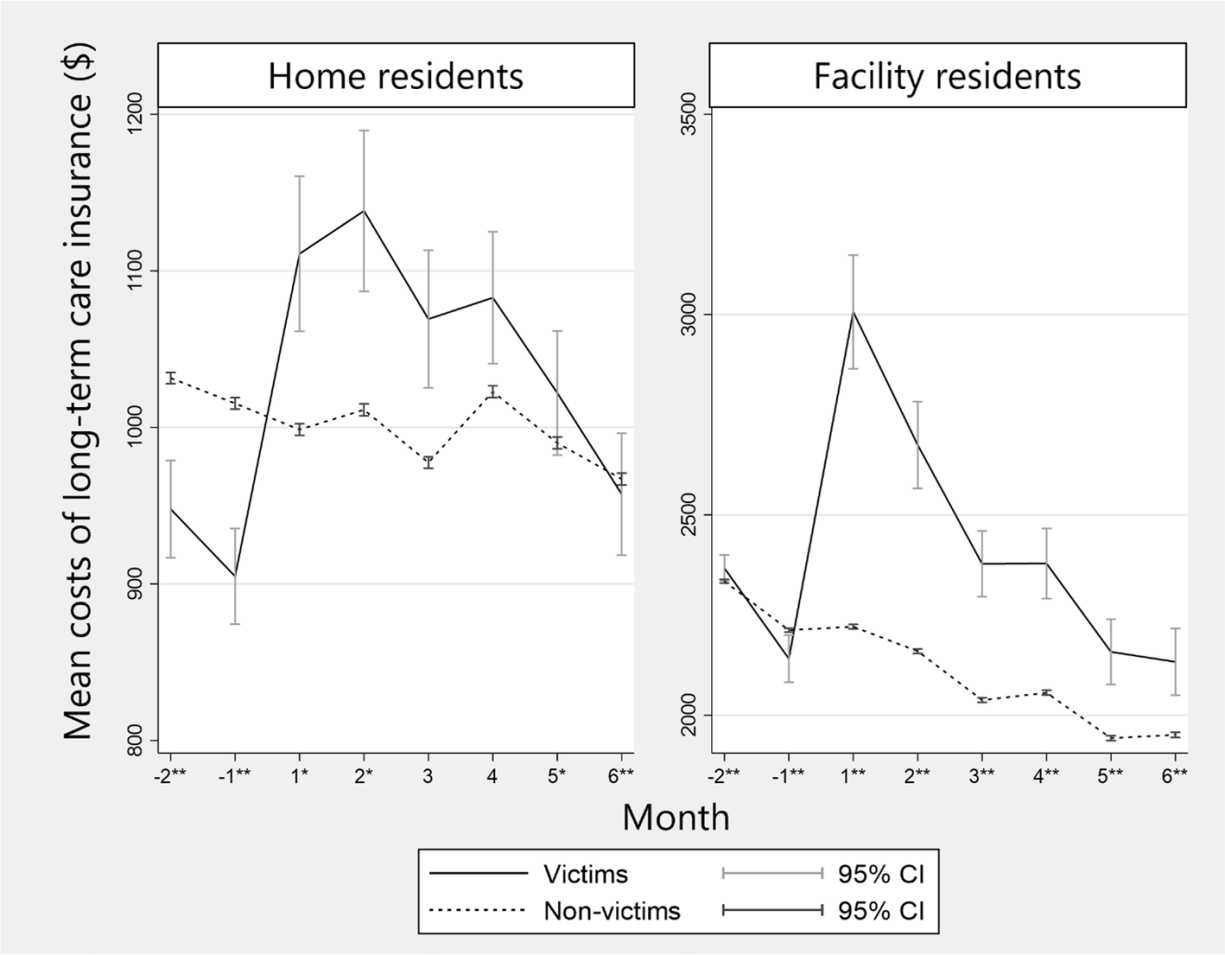
Mean costs for long-term care insurance system. Footnote: *: *P* value is < 0.05 by Wilcoxon rank-sum test. **: *P* value is < 0.001 by Wilcoxon rank-sum test. Month: month from the 2018 Japan Floods. CI: confidence interval

Figure 2 shows the mean costs for LTCI services for the period from 2 months before to 6 months after the disaster. Victims in home residents and facility residents increased the cost after the disaster (*p* < 0.001). For the home victims, the increase in the mean cost converged within the observation period. On the other hand, for facility victims, the increase had not converged during the observation period.

**Fig. 3:**
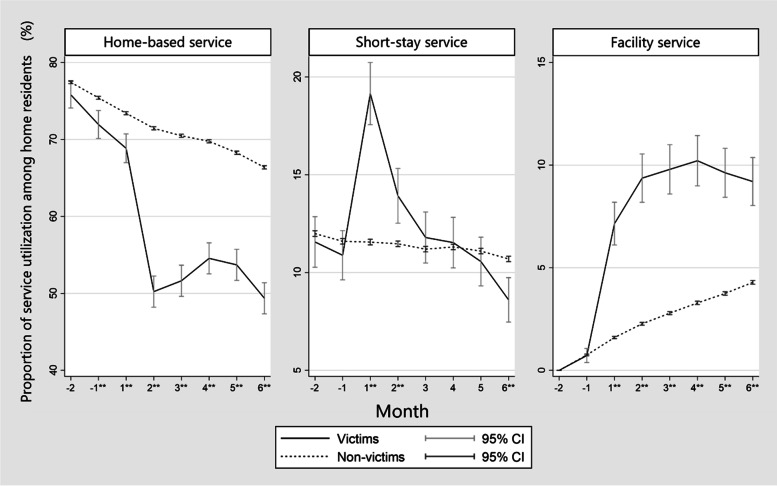
Proportion of service utilization among home residents. Footnote: **: P value is < 0.001 by Wilcoxon rank-sum test. Month: month from the 2018 Japan Floods. CI: confidence interval

Figure 3 shows the proportion of participants who used each LTCI service among home residents by month. Victims less used home-based services through all periods (*p* < 0.001). However, victims used more short-stay services until 2 months after the disaster (*p* < 0.001), and facility services for all months after the disaster (*p* < 0.001).

**Fig. 4:**
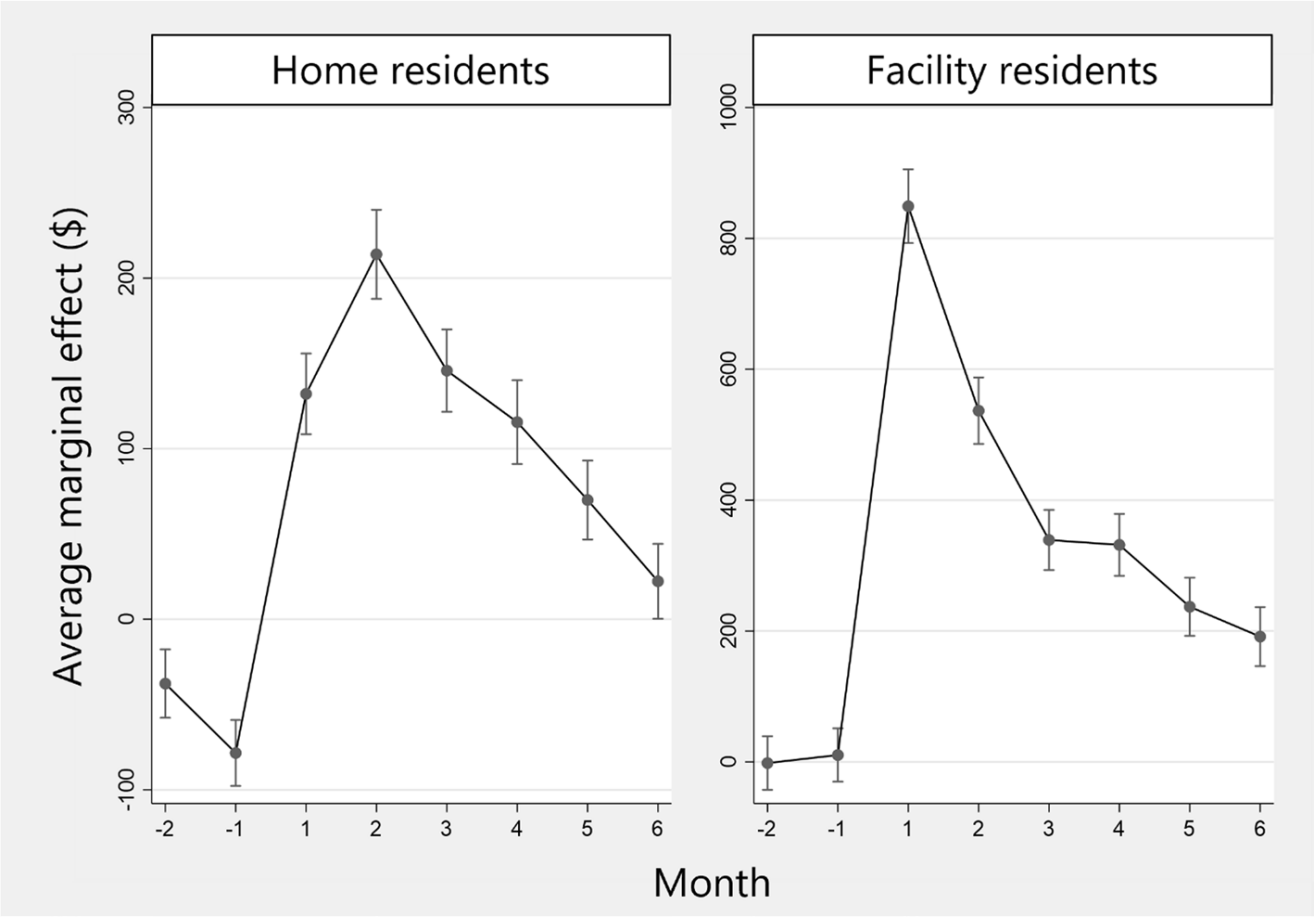
Average marginal effect on long-term care insurance costs of victims as the attributable costs from the disaster from the results of generalized estimating equations. Footnote: Average marginal effect on the utilization of the long-term care insurance costs of victims is the attributable cost due to the 2018 Japan Floods. We estimated the average marginal effect from the result of generalized estimating equations

We showed the results of GEEs on total costs of LTCI and estimated AME on the LTCI costs of victims as the attributable costs from the disaster (Fig. 4, Supplementary Table 2 [see Supplementary File 2] and Supplementary Table 3 [see Supplementary File 3]). Among home residents and facility residents, the attributable costs of the disaster increased during all the months after the disaster. The attributable cost for home residents at 2 months after was $214 (Standard Error (SE): 12, *p* < 0.001), which was the highest through the observation period. In comparison, among facility residents, the attributable cost immediately after the disaster increased by up to $850 (SE: 29, *p* < 0.001).

**Fig. 5:**
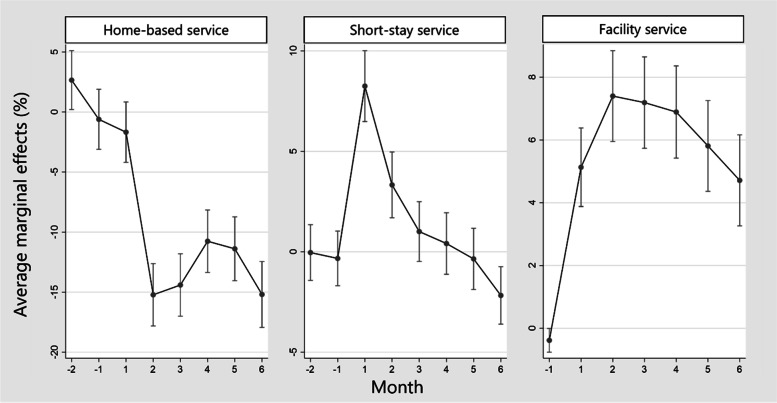
Average marginal effect on the utilization of long-term care insurance services of victims among home residents. Footnote: Average marginal effects on the utilization of long-term care insurance services of victims among home residents is the attributable relative risk due to the 2018 Japan Floods. We estimated the average marginal effect from the result of generalized estimating equations

As with cost, we estimated AME on the service utilization of home victims as the attributable risk from the disaster (Fig. 5, Supplementary Table 4 [see Supplementary File 4] and Supplementary Table 5 [see Supplementary File 5]). The AME of utilization for home-based service were lower among victims than non-victims from 2 months after the disaster. The lowest AME was − 15.2% (SE:1.3, *p* < 0.001) at 2 months after the disaster. On the other hand, the AME for short-stay service increased by up to 8.2% (SE: 0.9, *p* < 0.001) and the AME for the facility service increased by up to 7.4% (SE: 0.7, *p* < 0.001), respectively.

## Discussion

The monthly total costs for LTCI services of victims increased regardless of the residential environment. Details of changes amongst home residents include: 1) an increase in short-stay services and facility services and 2) a decrease of home-based services. Similarly, the cost for facility-victims increased after the disaster. These changes gradually diminished after 6 months.

The monthly total cost for LTCI services among home residents increased by up to $213.60 (SE: 13.3, *p* < 0.001) at 2 months after the disaster, and diminished gradually for a half-year, even though disuse months of LTCI services amongst victims were more than non-victims. After the GEJE, an ecological study showed that the total costs for LTCI services decreased in the affected area [30]. Earthquakes and a tidal waves cause extensive damage in a geographically consistent manner [45]. In comparison, areas impacted by floods were mainly near rivers or along slopes and unaffected areas were mixed even in the same municipality [46]. LTCI services in an unaffected area could complement services in an affected area where services become unavailable. Therefore, the duration of a full stop in the supply of LTCI services may be short. Increases in LTCI service use would have been supported by undamaged facilities or services. However, we cannot prove that such care needs truly arose caused by the disaster because the definition of victims was based on policy [34]. Since this policy gave affected people economic incentives, they might have been encouraged to use more LTCI services than non-victims and this should be considered when interpreting the results of this study.

Victims who had been using home-care services increased the utilization of short-stay services and facility services after the disaster. The highest AME of short-stay service was 8.2% (SE: 0.9, *p* < 0.001) and that of facility services increased up to 7.4% (SE: 0.7, *p* < 0.001). These victims and their families likely choose emergent use of these services because it was difficult to receive care at common shelters and the disaster decreased a family’s ability to provide care [47]. MHLW announced the abolition of the upper limit in use of short-stay services for the evacuation [48]. This announcement would encourage more use of short-stay services. To adapt to emergencies, facilities in disaster hazard zones should plan for a modifiable fixed number in accordance with the scale of the disaster to accept vulnerable victims. Meanwhile, health issues continue to require study for victims evacuated to LTCI facilities as few studies have examined such services in care facilities. Past studies showed that elderly persons evacuated to shelters had lower health outcomes including a higher mortality rate [49–51]. However, in evacuation to LTCI facilities, they may receive needed essential care. This could prevent their health decline, such as ADL and quality of life. Accordingly, prognosis should be examined in future studies. Based on the results here, the local government and service providers should organize facility allocation and estimate a fixed number of added patients that can be admitted to each facility in an emergency during future disasters.

Among home residents, the AME of home-based service continued to decrease from 2 months after the disaster, and the lowest AME was − 15.2% (SE: 1.3, *p* < 0.001). Since the beginning of the month when the disaster occurred was still before the actual damage, the utilization of home-based services of the first month would not have declined. This decline was caused by the disruption of transportation networks or utilities [10]. Because the disruption could have decreased home care capabilities, the use of short-stay services and facility services might have increased. After being admitted to a facility, it is often difficult to return elderly patients back home [52]. This situation delayed the recovery of home-based services.

The cost for facility-victims immediately increased after the disaster by $850.30 (SE: 28.8, *p* < 0.001) and gradually diminished over 6 months. An increase of facility-victims would represent evacuation to different facilities. Because of the emergency, some refugees were changed from a multi-bedroom to a private room. In addition, MHLW announced a special provision, which could claim the cost for a private room when multiple refugees used a single room together as a multi-bedroom [53]. This would cause an increase in the cost for facility-victims. Facility-victims who were usually at a low ADL level or cognitive function find it difficult to use common shelters. Accordingly, they had to evacuate to other facilities. In the GEJE, short- and long-term mortality increased among facility-victims that evacuated to different facilities [51]. This may have been due to a lack of readiness on the part of shelters to deal with victims as well as fragmentation from previous care providers [51]. Upon a disaster, the decision of whether facility-victims need to evacuate should be made considering usable resources [54].

This was the first study to examine the attributable cost of the 2018 Japan Floods on long-term care, using LTCI claim data that covered all LTCI service users. The LTCI comprehensive database reflects a public insurance system and covers almost all persons in Japan aged over 65 years old. Therefore, this study examined the overall effect of the disaster on LTCI system. The database is highly accurate as it is managed by the national government. The results of this study can estimate the elevated costs of LTCI and the change in service utilization of LTCI chronologically per the magnitude of the torrential rains and floods or the number of the victims. Based on this study, the government needs to plan care for the elderly as early as possible and respond quickly after a disaster occurs. Moreover, local government and service providers should organize facility allocation to prepare for a disaster and estimate a capacity for an emergency.

This study has several limitations. First, the LTCI database rarely has information about death and medical attendance, such as diseases, treatment and admission. Although there is no information in this database, we evaluated the quantitative changes in the costs and service utilization of LTCI as the population average using the GEEs. To estimate the effect of LTCI, this study could show the results with available resources. Moreover, since this database started to contain the information to link the National Database of Health Insurance Claims from March 2020, the study integrated both databases can be organized hereafter. Second, although we defined “victims” as LTCI service users who were exempted from out-of-pocket expenses after the disaster, some other persons were also given relief from out-of-pocket payments due to the disaster. However, we could not distinguish such persons from non-victims. Therefore, we risk underestimating the effect of the disaster. Third, the results of this study did not include the cost of informal care and resources which was not covered by LTCI system. As a future study, it is also important to investigate a specific population among victims and capture changes in informal care. Moreover, facility residents were fewer in victims. Generally, facility residents are older and more vulnerable than home residents. The risk of flooding and its adverse health impacts are unevenly distributed [15]. Because most facilities are built in areas less prone to natural disasters, the 2018 Japan Floods damaged mainly private homes and home residents. A similar trend is likely in future torrential rains and floods.

## Conclusions

The 2018 Japan Floods increased LTCI service costs for victims regardless of the residential environment. The increase gradually diminished over half-a-year. Considering the increased costs and utilization of care facilities according to the disaster scale, local governments and care providers should prepare for future floods and other emergencies.

## Supplementary Information

**Additional file 1: Supplementary Table 1.** Service Classification Codes (*service-kubun-code*).

**Additional file 2: Supplementary Table 2.** Results of Generalized Estimating Equations on Total Costs of Long-term Care Insurance System.

**Additional file 3: Supplementary Table 3.** Average Marginal Effect on Long-term Care Insurance Costs of Victims as Attributable Costs from the Disaster ($).

**Additional file 4: Supplementary Table 4.** Results of Generalized Estimating Equations on Service Utilization of Long-term Care Insurance System.

**Additional file 5: Supplementary Table 5.** Average Marginal Effects on Utilization of Long-term Care Insurance Services of Victims Among Home Residents (%).

## Data Availability

The data that support the findings of this study are available from the Ministry of Health, Labour and Welfare, but restrictions apply to the availability of these data, which were used under license for the current study, and so are not publicly available. Data are however available from the authors upon reasonable request and with permission of the Ministry of Health, Labour and Welfare.

## Abbreviations

AME: Average marginal effects
ADL: Activity of daily living
GEE: Generalized estimating equation
GEJE: Great East Japan Earthquake
LTCI: Long-term care insurance
MHLW: Ministry of Health, Labour and Welfare
SE: Standard error
WHO: World Health Organization
