# Supplementary material for: Effects of the 2018 Japan Floods on long-term care insurance costs in Japan: retrospective cohort study
Source: BMC Public Health. 2022 Feb 17;22:341. doi: 10.1186/s12889-022-12492-7 (PMC8855556; doi:10.1186/s12889-022-12492-7)
Supplement: Supplementary file 1 — Additional file 1: Supplementary Table 1. Service Classification Codes (service-kubun-code). [file 12889_2022_12492_MOESM1_ESM.docx]

Supplementary Table 1: Service Classification Codes (*service-kubun-code*)

| Service type | Service classification code |
| --- | --- |
| Home-based service | 11, 12, 13, 14, 15, 16, 62, 63, 64, 66, 71, 72, 73, 74, 76, 78 |
| Short-stay services | 21, 22, 23, 24, 25, 26, 27, 28, 38, 39 |
| Facility services | 32, 33, 35, 36, 37, 51, 52, 53, 54 |
