# Supplementary material for: Effects of the 2018 Japan Floods on long-term care insurance costs in Japan: retrospective cohort study
Source: BMC Public Health. 2022 Feb 17;22:341. doi: 10.1186/s12889-022-12492-7 (PMC8855556; doi:10.1186/s12889-022-12492-7)
Supplement: Supplementary file 2 — Additional file 2: Supplementary Table 2. Results of Generalized Estimating Equations on Total Costs of Long-term Care Insurance System. [file 12889_2022_12492_MOESM2_ESM.docx]

Supplementary Table 2: Results of Generalized Estimating Equations on Total Costs of Long-term Care Insurance System

| Covariates | | Home residents | | Facility residents | |
| --- | --- | --- | --- | --- | --- |
|  |  | Coef. | SE | Coef. | SE |
| Disaster status | Non-victims | Reference | | Reference | |
|  | Victims | -0.039** | 0.011 | -0.00044 | 0.009 |
| Month | -2 | Reference | | Reference | |
|  | -1 | 0.004* | 0.001 | -0.032** | 0.001 |
|  | 1 | -0.002** | 0.001 | -0.009** | 0.001 |
|  | 2 | 0.018** | 0.001 | -0.019** | 0.001 |
|  | 3 | -0.006** | 0.001 | -0.060** | 0.001 |
|  | 4 | 0.041** | 0.001 | -0.033** | 0.001 |
|  | 5 | 0.020** | 0.001 | -0.070** | 0.001 |
|  | 6 | 0.011** | 0.001 | -0.041** | 0.001 |
| Age | 40 - 65 y. | Reference | | Reference | |
|  | 65 - 74 y. | 0.001 | 0.006 | -0.039** | 0.007 |
|  | 75 - 84 y. | 0.015* | 0.006 | -0.055** | 0.006 |
|  | 85 - y. | 0.067** | 0.006 | -0.072** | 0.006 |
| Gender | Male | Reference | | Reference | |
|  | Female | 0.061** | 0.002 | 0.048** | 0.001 |
| Care level | Support need level 1 | Reference | | Reference | |
|  | Support need level 2 | 0.45** | 0.002 | 0.59** | 0.006 |
|  | Care need level 1 | 1.1** | 0.002 | 1.2** | 0.005 |
|  | Care need level 2 | 1.3** | 0.002 | 1.3** | 0.005 |
|  | Care need level 3 | 1.6** | 0.003 | 1.4** | 0.005 |
|  | Care need level 4 | 1.8** | 0.003 | 1.5** | 0.005 |
|  | Care need level 5 | 1.9** | 0.004 | 1.5** | 0.005 |
| Interaction term  between victims and month | -2 | Reference | | Reference | |
|  | -1 | -0.043** | 0.007 | 0.005 | 0.009 |
|  | 1 | 0.16** | 0.007 | 0.31** | 0.009 |
|  | 2 | 0.23** | 0.007 | 0.21** | 0.009 |
|  | 3 | 0.18** | 0.007 | 0.14** | 0.009 |
|  | 4 | 0.15** | 0.007 | 0.14** | 0.01 |
|  | 5 | 0.11** | 0.007 | 0.10** | 0.01 |
|  | 6 | 0.061** | 0.007 | 0.083** | 0.01 |

Footnote

Month: month from the 2018 Japan Floods

Coef.: Coefficient

SE: standard error

*: P value is < 0.05.

**: P value is < 0.001.
