# Supplementary material for: Effects of the 2018 Japan Floods on long-term care insurance costs in Japan: retrospective cohort study
Source: BMC Public Health. 2022 Feb 17;22:341. doi: 10.1186/s12889-022-12492-7 (PMC8855556; doi:10.1186/s12889-022-12492-7)
Supplement: Supplementary file 3 — Additional file 3: Supplementary Table 3. Average Marginal Effect on Long-term Care Insurance Costs of Victims as Attributable Costs from the Disaster ($). [file 12889_2022_12492_MOESM3_ESM.docx]

Supplementary Table 3: Average Marginal Effect on Long-term Care Insurance Costs of Victims as Attributable Costs from the Disaster ($)

| Victims  (Reference = non-victims) | Month | Home residents | | Facility residents | |
| --- | --- | --- | --- | --- | --- |
|  |  | AME | SE | AME | SE |
|  | -2 | -37.5** | 10.1 | -1.0 | 20.9 |
|  | -1 | -77.9** | 9.8 | 11.4** | 20.8 |
|  | 1 | 131.8** | 12.0 | 850.3** | 28.8 |
|  | 2 | 213.6** | 13.3 | 537.9** | 25.9 |
|  | 3 | 145.9** | 12.3 | 340.0** | 23.5 |
|  | 4 | 116.4** | 12.6 | 332.5** | 24.1 |
|  | 5 | 70.5** | 11.9 | 238.1** | 22.7 |
|  | 6 | 22.4* | 11.3 | 192.5** | 23.0 |

Footnote

AME: average marginal effect

SE: standard error

Month: month from the 2018 Japan Floods

*: P value is <0.05.

**: P value is <0.001.
