# Supplementary material for: Effects of the 2018 Japan Floods on long-term care insurance costs in Japan: retrospective cohort study
Source: BMC Public Health. 2022 Feb 17;22:341. doi: 10.1186/s12889-022-12492-7 (PMC8855556; doi:10.1186/s12889-022-12492-7)
Supplement: Supplementary file 4 — Additional file 4: Supplementary Table 4. Results of Generalized Estimating Equations on Service Utilization of Long-term Care Insurance System. [file 12889_2022_12492_MOESM4_ESM.docx]

Supplementary Table 4: Results of Generalized Estimating Equations on Service Utilization of Long-term Care Insurance System

| Covariates | | Home-based service | | Short-stay service | | Facility service | |
| --- | --- | --- | --- | --- | --- | --- | --- |
|  |  | Coef. | SE | Coef. | SE | Coef. | SE |
| Disaster status | Non-victims | Reference | | Reference | | Reference | |
|  | Victims | 0.034* | 0.016 | -0.004 | 0.063 | -0.55 | 0.277 |
| Month | -2 | Reference | | Reference | |  | |
|  | -1 | -0.003** | 0.0004 | -0.012* | 0.004 | Reference | |
|  | 1 | -0.015** | 0.001 | -0.004 | 0.005 | 0.71** | 0.017 |
|  | 2 | -0.026** | 0.001 | 0.002 | 0.005 | 1.0** | 0.019 |
|  | 3 | -0.030** | 0.001 | -0.021** | 0.005 | 1.2** | 0.020 |
|  | 4 | -0.032** | 0.001 | -0.008 | 0.005 | 1.4** | 0.021 |
|  | 5 | -0.039** | 0.001 | -0.018* | 0.005 | 1.5** | 0.021 |
|  | 6 | -0.049** | 0.001 | -0.039** | 0.006 | 1.6** | 0.021 |
| Age | 40 - 65 y. | Reference | | Reference | | Reference | |
|  | 65 - 74 y. | -0.005 | 0.009 | 0.18** | 0.048 | 0.62** | 0.16 |
|  | 75 - 84 y. | -0.004 | 0.009 | 0.38** | 0.047 | 1.2** | 0.16 |
|  | 85 - y. | -0.001 | 0.009 | 0.67** | 0.047 | 1.5** | 0.16 |
| Gender | Male | Reference | | Reference | | Reference | |
|  | Female | -0.007* | 0.003 | 0.11** | 0.013 | 0.13** | 0.028 |
| Care level | Support need level 1 | Reference | | Reference | | Reference | |
|  | Support need level 2 | 0.025* | 0.010 | 0.41** | 0.032 | 0.30* | 0.131 |
|  | Care need level 1 | 0.84** | 0.011 | 1.3** | 0.033 | 2.0** | 0.12 |
|  | Care need level 2 | 0.82** | 0.011 | 1.7** | 0.032 | 2.3** | 0.12 |
|  | Care need level 3 | 0.76** | 0.011 | 2.1** | 0.033 | 3.0** | 0.12 |
|  | Care need level 4 | 0.67** | 0.012 | 2.1** | 0.034 | 3.4** | 0.12 |
|  | Care need level 5 | 0.65** | 0.013 | 2.1** | 0.036 | 3.4** | 0.12 |
| Interaction term  between victims and month | -2 | Reference | | Reference | |  | |
|  | -1 | -0.042** | 0.006 | -0.027 | 0.039 | Reference | |
|  | 1 | -0.056** | 0.007 | 0.55** | 0.057 | 1.9** | 0.35 |
|  | 2 | -0.26** | 0.015 | 0.26** | 0.063 | 1.9** | 0.36 |
|  | 3 | -0.25** | 0.015 | 0.090 | 0.064 | 1.7** | 0.36 |
|  | 4 | -0.19** | 0.014 | 0.039 | 0.064 | 1.6** | 0.37 |
|  | 5 | -0.20** | 0.015 | -0.028 | 0.067 | 1.4** | 0.37 |
|  | 6 | -0.27** | 0.017 | -0.21* | 0.076 | 1.2* | 0.37 |

Footnote

Month: month from the 2018 Japan Floods

Coef.: Coefficient

SE: standard error

*: P value is <0.05.

**: P value is <0.001.
