# Supplementary material for: Effects of the 2018 Japan Floods on long-term care insurance costs in Japan: retrospective cohort study
Source: BMC Public Health. 2022 Feb 17;22:341. doi: 10.1186/s12889-022-12492-7 (PMC8855556; doi:10.1186/s12889-022-12492-7)
Supplement: Supplementary file 5 — Additional file 5: Supplementary Table 5. Average Marginal Effects on Utilization of Long-term Care Insurance Services of Victims Among Home Residents (%). [file 12889_2022_12492_MOESM5_ESM.docx]

Supplementary Table 5: Average Marginal Effects on Utilization of Long-term Care Insurance Services of Victims Among Home Residents (%)

| Month | Home-based service | | Short-stay service | | Facility service | |
| --- | --- | --- | --- | --- | --- | --- |
|  | AME | SE | AME | SE | AME | SE |
| -2 | 2.7 | 1.3 | -0.04 | 0.7 |  | |
| -1 | 0.6 | 1.3 | -0.3 | 0.7 | -0.4* | 0.2 |
| 1 | -1.7 | 1.3 | 8.2** | 0.9 | 5.1** | 0.6 |
| 2 | -15.2** | 1.3 | 3.3** | 0.8 | 7.4** | 0.7 |
| 3 | -14.4** | 1.3 | 1.0 | 0.8 | 7.2** | 0.7 |
| 4 | -10.8** | 1.3 | 0.4 | 0.8 | 6.9** | 0.7 |
| 5 | -11.4** | 1.4 | -0.4 | 0.8 | 5.8** | 0.7 |
| 6 | -15.2** | 1.4 | -2.2* | 0.7 | 4.7** | 0.7 |

Footnote

AME: average marginal effect

SE: standard error

Month: month from the 2018 Japan Floods

*: P value is <0.05.

**: P value is <0.001.
